# Personalized modeling of gut microbiome metabolism throughout the first year of life

**DOI:** 10.1038/s43856-024-00715-4

**Published:** 2024-12-30

**Authors:** Rola Shaaban, Susheel Bhanu Busi, Paul Wilmes, Jean-Louis Guéant, Almut Heinken

**Affiliations:** 1 Inserm UMRS 1256 NGERE, University of Lorraine, Nancy, France; 2 Luxembourg Centre for Systems Biomedicine, University of Luxembourg, Esch-sur-Alzette, Luxembourg; 3 UK Centre for Ecology and Hydrology, Wallingford, Oxfordshire, UK; 4 Department of Life Sciences and Medicine, Faculty of Science, Technology and Medicine, University of Luxembourg, Esch-sur-Alzette, Luxembourg; 5 National Center of Inborn Errors of Metabolism, University Regional Hospital Center of Nancy, Nancy, France; 6 Present Address: Nantes University, Nantes, France

**Keywords:** Dynamical systems, Microbiome

## Abstract

**Background:**

Early-life exposures including diet, and the gut microbiome have been proposed to predispose infants towards multifactorial diseases later in life. Delivery via Cesarian section disrupts the establishment of the gut microbiome and has been associated with negative long-term outcomes. Here, we hypothesize that Cesarian section delivery alters not only the composition of the developing infant gut microbiome but also its metabolic capabilities. To test this, we developed a metabolic modeling workflow targeting the infant gut microbiome.

**Methods:**

The AGORA2 resource of human microbial genome-scale reconstructions was expanded with a human milk oligosaccharide degradation module. Personalized metabolic modeling of the gut microbiome was performed for a cohort of 20 infants at four time points during the first year of life as well as for 13 maternal gut microbiome samples.

**Results:**

Here we show that at the earliest stages, the gut microbiomes of infants delivered through Cesarian section are depleted in their metabolic capabilities compared with vaginal delivery. Various metabolites such as fermentation products, human milk oligosaccharide degradation products, and amino acids are depleted in Cesarian section delivery gut microbiomes. Compared with maternal gut microbiomes, infant gut microbiomes produce less butyrate but more L-lactate and are enriched in the potential to synthesize B-vitamins.

**Conclusions:**

Our simulations elucidate the metabolic capabilities of the infant gut microbiome demonstrating they are altered in Cesarian section delivery at the earliest time points. Our workflow can be readily applied to other cohorts to evaluate the effect of feeding type, or maternal factors such as diet on host-gut microbiome inactions in early life.

## Abstract (web summary)

Shaaban et al. undertake personalized metabolic modeling of the infant gut microbiome during the first year of life. The gut microbiome of infants delivered through Cesarian section has reduced metabolic capabilities compared with that of vaginally delivered infants at early time points, and infant gut microbiomes are enriched in B-vitamin biosynthesis compared with adult gut microbiomes.

## Plain-language summary

Trillions of microorganisms live in the digestive system of humans, with those within the intestine being described as the intestinal microbiome. Intestinal microbes perform important metabolic functions such as digestion of the diet (e.g., breast milk) and production of metabolites such as B-vitamins. Birth via Cesarian section disrupts the establishment of the gut microbiome. Here, we evaluate the effect of birth mode on microbiome metabolic functions during the first year of life. Computational metabolic models were built for a cohort of mothers and infants, with each model representing the individual’s unique microbiome. Microbiomes from infants delivered by Cesarian section had perturbed metabolic functions early in life but became comparable to those in vaginally delivered infants later in life. Moreover, the metabolic functions present in infant gut microbiomes differed from those in maternal gut microbiomes. This information could be a useful starting point for further research to improve the intestinal microbiome of babies born by Cesarian section.

## Introduction

The human gut microbiome plays an important role in human health and well-being^1^. Changes in the composition and function of the gut microbiome have been implicated in noncommunicable diseases, including cardiometabolic diseases, neurodevelopmental diseases, and allergies^2^. During the first year of life, the gut microbiome performs essential functions such as maturation of the immune system, digestion of the diet, synthesis of amino acids and vitamins, and protection against pathogens^1,3^. Initial colonization of the gastrointestinal tract occurs at birth, and drastic changes in composition and diversity occur during the first year of life^4^. The infant gut microbiome is influenced by a variety of factors including diet, geography, mode of delivery, infection, feeding type (formula versus breastfed), and medication^5^. Specifically, breast milk shapes the composition of the infant gut microbiome as it contains large amounts of oligosaccharides (human milk oligosaccharides, HMOs) that are selectively utilized by beneficial gut microbes and promote their growth^6^. Hence, they aid in resisting colonization of pathogens, and in the maturation of the immune system by promoting short-chain fatty acid production^6^. Members of the Bifidobacterium and Bacteroides genera are especially adept at using HMOs as carbon sources^6^, but HMO utilization has also been shown in members of the Bacilliales^6^ and Clostridiales^7^ orders as well as the Verucomicrobia phylum^8^.

The mode of delivery also impacts the early gut microbiome. Vaginally delivered (VD) infants receive maternal microbes via the fecal-oral route, while this transmission is disrupted in infants born via Cesarian section delivery (CSD)^5^. The gut microbiome of VD infants is first colonized by facultative anaerobes such as Escherichia, followed by Bifidobacterium and Bacteroides species shortly after^9,10^. In contrast, those born through CSD acquire species more often associated with the hospital environment such as *Staphylococcus epidermis* and *Bacteroides fragilis*^9^. Short-term risks of CSD include reduced diversity of the gut microbiome, impaired transmission of bacterial strains from mother to newborn, microbiome-related functional deficits, and predisposition to colonization by opportunistic pathogens, including those carrying antimicrobial resistance genes^9,11^. Delivery by the Cesarian section has been associated with a higher risk of allergies, asthma, obesity, and neurodevelopmental disorders^3,12^.

The Developmental Origins of Health and Disease (DOHaD) hypothesis, referred to as fetal programming, states that environmental exposures, such as nutrients, chemicals, and drugs, during vulnerable developmental stages can permanently program changes in offspring organ structure and function toward the development of noncommunicable diseases^13,14^. Recently, the gut microbiome has been proposed as an exposure affecting early-life development^5^. Maternal obesity during pregnancy can predispose infants to obesity later in life^15^, which may be mediated by the maternal gut microbiota^5^. In animal models, obesity could be transferred from mothers to offspring mediated by diet and the gut microbiota^16^, however, evidence in humans for the transmittal of obesity via the gut microbiome remains to be established. A key mechanism through which early-life environmental exposures affect predisposition towards metabolic diseases later in life, and health outcomes can be transmitted across generations, is epigenetic regulation^17^. Gut microbes produce short-chain fatty acids, which are metabolized by the human host and directly influence gene regulation through at least four independent mechanisms^18^. For instance, butyrate acts as a histone deacetylase inhibitor^18^. Consistently, in animal models, the gut microbiome has been shown to modulate global histone acetylation and methylation profiles in multiple host tissues in a diet-dependent manner^19^, an obesogenic gut microbiota combined with a high-fat diet reprogrammed the intestinal epigenome^20^, and a long-term high-fat diet led to alterations of histone methylation and acetylation in epithelial cells^21^. Hence, the gut microbiome has been proposed to contribute to metabolic programming in early life via epigenome-mediated mechanisms^22^. Besides short-chain fatty acids, gut microbes also produce vitamins, including folate and B12 which play a key role in epigenetic regulation^17^, and a variety of other bioactive metabolites that modulate the regulation of host metabolism during the first year of life^23^. Hence, delivery mode as well as other factors influencing infant gut microbiome composition (e.g., maternal diet and feeding^5^) may also affect microbial metabolites and their crosstalk with host metabolism.

To link the gut microbiome and its impact on bioactive metabolites in the fecal or blood metabolome beyond correlations, mechanistic systems biology models are needed^24^. Constraint-based reconstruction and analysis (COBRA) have emerged as an attractive mechanistic systems biology approach for modeling the human microbiome^24^. Briefly, COBRA employs genome-scale reconstructions of metabolism that are built in a bottom-up manner and curated through manual efforts based on genomic, biochemical, and physiological knowledge^25^. A resource of 773 curated genome-scale reconstructions of the human gut microbiome, deemed AGORA^26^, has enabled the strain-and molecule-resolved prediction of metabolic differences between patients and controls through dedicated modeling pipelines^27,28^. An expansion of AGORA, accounting for 7302 strains and deemed AGORA2, has been published recently^29^. Previous studies have used COBRA modeling to elucidate metabolic mechanisms in the infant’s gut. Metagenomic and nutritional data from a cohort of 13 infants at five-time points were integrated into a reference metabolic network and used to predict gut microbial metabolic patterns before and after the introduction of solid foods^30^. Another study used 15 reconstructions from the AGORA resource to build a spatiotemporal model of the infant colon^31^. The model predicted cross-feeding between members of the infant’s gut and could reproduce the switch from facultative anaerobes to an anaerobic environment dominated by bifidobacteria^31^. A follow-up study introduced degradation of the HMO 2-fucosyllactose into the spatiotemporal model revealing that 2-fucosyllactose promoted butyrate production through cross-feeding mechanisms^32^. In another recent study, a high-quality genome-scale reconstruction of Bifidobacterium *longum* subsp. infantis was built using the AGORA reconstruction as a template and accounting for HMO degradation^33^.

Here, we aim to investigate through metabolic modeling if the birth mode has an impact on the metabolic capabilities of the infant gut microbiome throughout the first year of life. Our hypothesis is that the known disruption of the establishment of the gut microbiome in CSD, resulting in an altered composition, would also result in altered microbial metabolic functions. To test this hypothesis, we use metagenomic data from gut microbiomes of infants born by VD or CSD at four-time points, as well as maternal gut microbiome samples from the same cohort, and build a personalized genome-scale community model of each sample at each time point. Our results demonstrate that at the earliest time points, the gut microbiome of infants delivered through Cesarian section is depleted in metabolic functions including human milk oligosaccharide degradation and bile acid transformation, while later in the first year of life, metabolic functions in CSD and VD gut microbiomes become comparable. We also show that the gut microbiome becomes more functionally diverse during the first year of life. Finally, we compare the metabolic potential of infant and adult gut microbiomes and show that infant gut microbiomes produce different levels of fermentation products than those of adults and are enriched in the potential to synthesize B vitamins.

## Materials and methods

### Description of the cohort

Metagenomic sequencing data from 20 infants at 5 days, 1 month, 6 months, and 1 year was retrieved from samples from the COSMIC cohort^4,11,34^. Briefly, the COSMIC (Colonization, succession, and evolution of the gut microbiome from birth to infancy) cohort was recruited from women giving birth at the Centre Hospitalier de Luxembourg (CHL) starting in 2012. The collected information on infants included delivery mode, birth weight, gestational age, sex, body length, weight, and feeding regime. More information is given in Ref. ^34^. For a subset of infants 16S rRNA sequencing data from mothers had been published in Ref. ^34^. Maternal gut microbiome data for 13 samples was retrieved from https://pmc.ncbi.nlm.nih.gov/articles/instance/6269548/bin/41467_2018_7631_MOESM6_ESM.xlsx.

### Ethics statement

As described previously^4^, written informed consent was obtained before specimen collection from all enrolled mothers after a detailed consultation. All aspects of recruitment, as well as the collection, handling, processing, and storing of samples and data, were approved by the Luxembourg ethics board, the Comité national d’éthique de Recherche, under reference number 201110/06, and by the Luxembourg National Commission for Data Protection under reference number A005335/R000058. As no new raw data was generated in the present study, additional approval by the ethics board was not necessary.

### Mapping of metagenomic sequencing data

Metagenomic sequencing was performed on the NextSeq500 (Illumina) instrument using 2 × 150 bp read length at the LCSB Sequencing Platform and processed using the metagenomic workflow of the Integrated Meta-omics Pipeline (IMP)^35^. Kraken2^36^ was used to map the reads against the Struo2 database^37^ and to generate files of species-level taxonomic assignment and abundance of metagenomics data. In total, 8882 taxa had been determined through the Kraken pipeline. After excluding unclassified species and those with low abundance, 617 species remained, of which 328 were name-matched to species present in the AGORA2^29^ resource through a custom script.

### In silico simulations

All simulations were performed in MATLAB version R2020b (Mathworks, Inc., Natick, MA, USA) using IBM CPLEX (IBM, Inc.) as the linear programming solver. The simulations relied on functions implemented in the COBRA Toolbox^38^.

### Generation of genome-scale reconstructions

Curated genome-scale reconstructions for 289 species not in AGORA2 were generated as follows. The KBase online framework^39^ was used to build draft reconstructions. Reference genomes for one strain per species were retrieved using the Narrative interface. Reference genomes were annotated using the “Annotate Multiple Microbial Assemblies with RASTtk-v1.073” app. Draft reconstructions were generated through the “Build Multiple Metabolic Models” app. Draft reconstructions were downloaded in SBML format using “Bulk Download Modeling Objects”. The dedicated Narrative can be found at https://narrative.kbase.us/narrative/111089.

The 289 generated draft reconstructions were then refined through the DEMETER^40^ pipeline. First, experimental data was collected for 28 named species as described previously^29^. The collected experimental data and taxonomic information for the 289 reconstructed strains were used as inputs for DEMETER. The resulting 289 refined genome-scale reconstructions are described in Supplementary Data 1.

### Formulation of HMO degradation module

Literature and database searches were performed for the utilization of human milk oligosaccharides (HMOs) by the gut microbiome. Species- and strain-specific utilization capabilities and transport and enzyme mechanisms were identified from relevant articles^6^. A total of 243 HMO-utilizing AGORA2 strains from 31 species were identified based on 24 peer-reviewed papers. A further seven strains were supplemented with pathways for monosaccharides present in HMOs. All identified species- and strain-specific HMO degradation capabilities are shown in Supplementary Data 2 with the corresponding references. The structures of 38 HMOs metabolites and HMOs degradation products were retrieved from 15 peer-reviewed papers and the Human Metabolome Database^41^. Reactions constituting the pathway were formulated based on transport and enzymatic mechanisms retrieved from reviewed papers (Supplementary Data 3a, b). An established protocol for high-quality genome-scale reconstruction^42^ was followed to ensure that the reactions and metabolites followed the nomenclature and quality standards in the field. Through the rBioNet tool^43^, it was ensured that the reactions were mass-and charge-balanced. The manually identified and formulated reactions and metabolites were added to the appropriate AGORA2 reconstructions through the custom MATLAB scripts runRefinement.m and HMOGapfill.m, resulting in the addition of 16.12 ± 11.03 reactions on average.

### Diet formulation

To appropriately contextualize gut microbiome models, we defined breast milk- and formula-feeding-based diets at 5 days, 1 month, 6 months, and 1 year of age. A literature search resulting in 10 consulted peer-reviewed articles was performed for the metabolic components of breast milk and their concentration. The composition of the formula diet was based on Similac Pro-Advance Infant Formula Powder. Components and concentrations were retrieved from https://www.similac.com/products/baby-formula/pro-advance-powder.html.

Metabolite concentrations were converted into fluxes (mmol/person/day) while assuming a daily milk consumption of 480 mL at 5 days, 720 ml at 1 month, 1260 ml at 6 months, and 1320 at 1 year. For diets at 6 months and 1 year of age, additional consumption of solid food was assumed. Fluxes corresponding to baby food items were retrieved from the Diet Designer tool on the Virtual Metabolic Human database^44^. For 6 months, 50 g of apple sauce strained and 50 g of chicken soup were assumed as consumed solid foods. For 12 months, 1/2 cup of bananas with apples and pears = 112 g, 1/2 baby food yogurt = 122.5 g, and 1/2 cup of chicken soup = 120 g were assumed. The uptake constraints for each formulated diet are listed in Supplementary Data 4.

### Personalized simulations

To build personalized microbiome models for infant gut and maternal gut samples, the mgPipe workflow in the Microbiome Modeling Toolbox v2.0^27^ tool was used, which takes relative organism abundance data as the input. As the taxonomic information obtained from Kraken was on the species level, the function createPanModels was used to create pan-species models from the expanded AGORA2 version (AGORA2 + 289). Pan-species models consist of all unique reactions present in at least one strain reconstruction as well as a merged version of all strain reconstructions’ biomass objective functions. Compartmentalized gut microbiome community models were built with the normalized species-level abundance data and pan-species models as the inputs. Briefly, all pan-species models corresponding to species present in a sample were joined in silico through a shared environment representing the intestinal lumen, which enables metabolite flow between species. The intake of dietary nutrients, and the excretion of metabolic products were enabled through exchange and transport reactions for dietary metabolites and a separate set of exchange and transport reactions representing fecal secretion. A community biomass function was added in which the normalized relative abundances served as stoichiometric parameters, hence enforcing growth ratios between species accordingly.

Each gut microbiome model was subsequently parameterized with the appropriate diets described in the previous section according to sample metadata on infants (Supplementary Data 5). One infant receiving combined breast and formula feeding was assumed to receive the breastfeeding diet in silico. Diets were converted to uptake fluxes through a dedicated function (convertVMHDiet2AGORA.m). Reaction abundance, reaction presence, subsystem abundance, and net secretion fluxes were determined by running the main mgPipe workflow (initMgPipe.m). Briefly, reaction abundances reflect for each microbiome model and each unique reaction the total relative abundances of species that carry the reaction. Subsystem abundances represent the reaction abundances summarized on the metabolic subsystem level for each microbiome model. The computed net secretion fluxes represent the maximal quantitative metabolite production flux that can be achieved by the microbiome community, subtracted by dietary uptake of metabolites.

For the comparison between infant and maternal samples, a default Average European diet was used for consistency. Net secretion fluxes for infant gut microbiomes and maternal gut microbiomes were computed. For 12 metabolites, species-level contributions were additionally computed through the predictMicrobeContributions function. Species-level contributions represent the maximal secretion flux of an individual pan-species model for a metabolite of interest. Note that due to the steady-state assumption resulting in multiple possible solutions, not every species will maximally secrete the metabolite in each possible flux distribution. For better visibility, the average species to metabolite contribution was calculated for each stratification group and then summarized on the genus level.

### Statistics and reproducibility

Statistical analyses by the Wilcoxon rank sum test and calculation of Spearman correlations were performed in MATLAB. In either case, correction for multiple testing by the Benjamini–Hochberg procedure was subsequently performed. All analyses can be reproduced using the provided code.

## Results

**Fig. 1:**
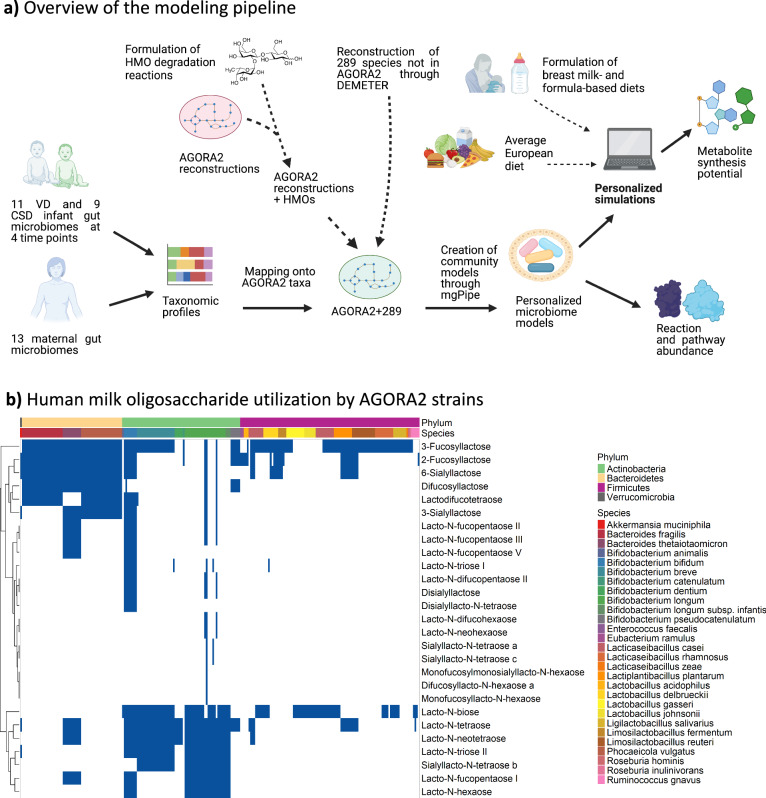
Overview of the modeling and analysis workflow and performed refinement. **a** Schematic overview of the personalized microbiome model generation and analysis pipeline. Shown is the formulation of the HMO degradation module, the construction and parameterization of personalized microbiome models, and the prediction of metabolite fluxes and reaction abundance profiles. Created in https://BioRender.com. **b** Human milk oligosaccharide utilization by AGORA2 strains represented in the expanded genome-scale reconstructions. Rows represent HMO metabolites, and columns represent all 243 AGORA2 reconstructions with at least one known HMO utilization capability annotated by species and phylum. Blue = strain can degrade this HMO, white = strain cannot degrade this HMO.

We developed a pipeline for modeling the metabolic capabilities of the infant gut microbiome during the first year of life (Fig. 1a, “Methods”). We used species-level abundances that had been inferred based on contaminant-free, high-resolution metagenomic sequencing data from the gut microbiomes of 20 infants, including 11 vaginally delivered (VD) and nine delivered through Cesarian section (CSD), at 5 days, 1 month, 6 months, and 1 year of age^11^. We reconstructed personalized microbiome models for the 20 infants and four-time points (“Methods”). Moreover, 13 maternal gut microbiome models were constructed (“Methods”). Infant microbiome models were appropriately contextualized with a breast milk or formula feeding-based diet. Contextualized microbiome models were interrogated in simulations as described previously^45^, yielding community-level relative reaction and subsystem abundances and net secretion potential for metabolites, as well as species-level contributions to metabolites of interest. Ultimately, the analysis yielded structural and functional features of gut microbiomes that differed between VD and CSD at each time point, in infants between time points, and between infants and adults.

### Literature-driven formulation of HMO degradation pathways

To account for gut microbial degradation of HMOs, we first performed a literature search of HMO degradation mechanisms and their distribution across human microbes yielding 19 species known to degrade at least one HMO or a simple sugar present in HMOs (Supplementary Data 2). Based on information from 14 peer-reviewed articles, we formulated an HMO degradation module accounting for 34 metabolites and 78 reactions, encompassing 28 HMOs and seven simple sugars and disaccharides (“Methods”, Supplementary Data 3a, b). HMO metabolites and degradation reactions were added to the appropriate AGORA2 reconstructions, adding on average 16.12 ± 11.03 reactions to 250 AGORA2 reconstructions. While previous works have reconstructed 2-fucosyllactose degradation^32^, and the HMO utilization by B. *longum* subsp. infantis^33^, the present work, to our knowledge is the most comprehensive HMO reconstruction to date in terms of captured HMO structures and species. The largest capacity for HMO degradation was found in *Bifidobacterium longum* subsp. infantis, followed by other Bifidobacterium sp., (Fig. 1b), in agreement with the experimental evidence^6^. Other gut bacteria with HMO degradation capability included Bacteroides sp., *Phocaeicola vulgatus*, Roseburia sp., *Akkermansia muciniphila*, and several species formerly assigned to the Lactobacillus genus (Fig. 1b).

### Generation and interrogation of personalized gut microbiome models

We mapped species-level abundances determined for reference genomes based on the metagenomic data from the 20 subjects at four time points^11^ to the 1744 species found in the AGORA2^29^ resource, and reconstructed 289 species not present in AGORA2 through the DEMETER^40^ pipeline (“Methods”, Supplementary Data 1). The reconstructions from AGORA2 together with the newly created 289 strain-level reconstructions, were deemed AGORA2 + 289. Using the species-level abundances that had been determined previously^11^ as the input data for the mgPipe pipeline^27^ (“Methods”), 71 personalized microbiome models were built by joining the appropriate AGORA2 + 289 genome-scale reconstructions into a compartmentalized community model (“Methods”, Supplementary Data 5). On average, the personalized infant gut microbiome models derived from AGORA2 + 289 encompassed 47.90 ± 19.76 species, 76,028.76 ± 24,459.82 reactions, and 67,915.83 ± 22,158 metabolites.

### Functional characterization of infant gut microbiomes

**Fig. 2:**
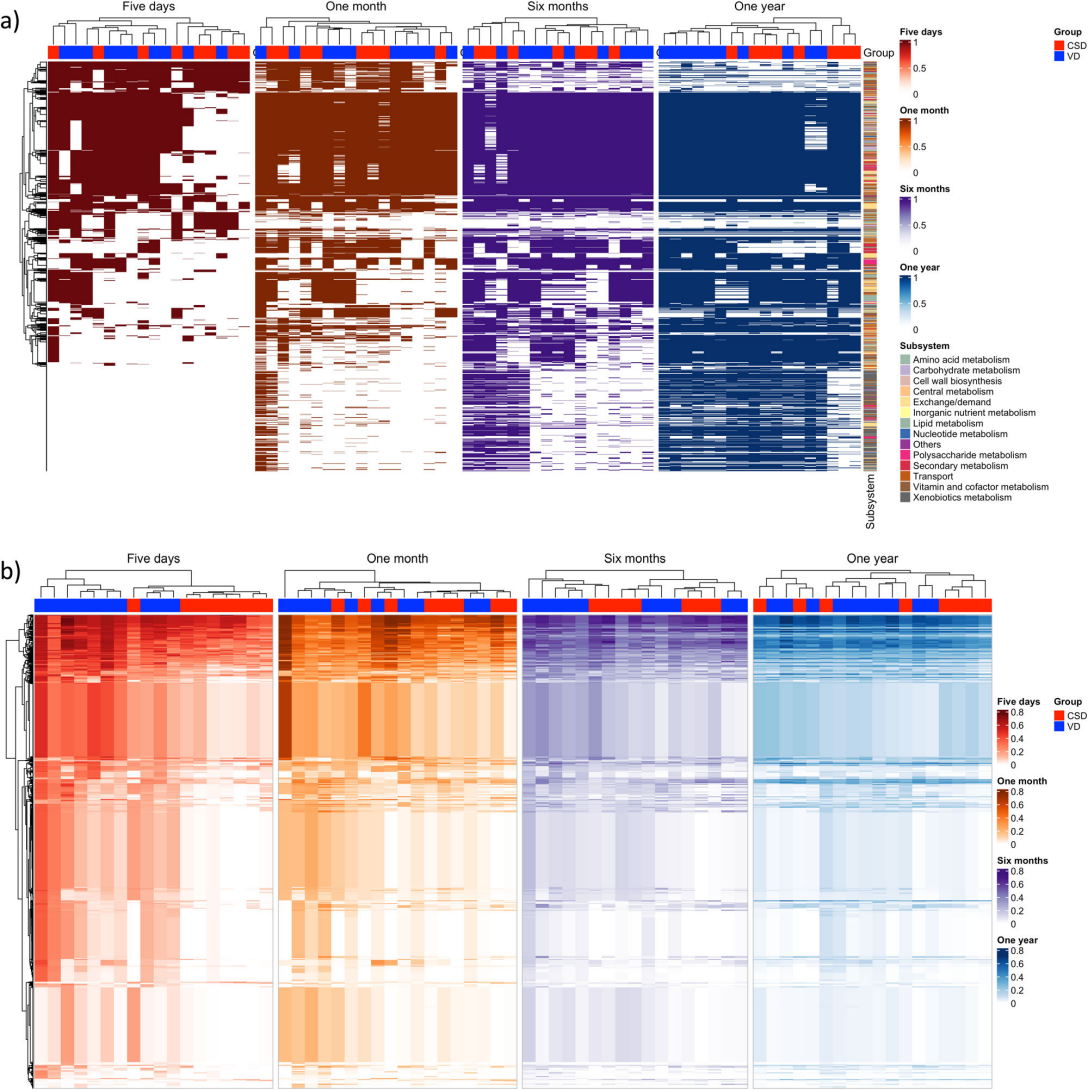
Significantly different features, and metabolic network structure of infant microbiomes plotted by time point and birth mode. **a** Absolute presence of reactions that differed significantly between time points after correction for multiple tests. Colored = reaction present, white = reaction absent. **b** Relative abundance of reactions that differed significantly between VD and CSD. Shown are reactions that were significantly different after correction for multiple testing for at least one-time point. All predicted reaction abundances are shown in Supplementary Data 6. *n* = 20 infant gut microbiome samples.

We next evaluated the functional differences between VD and CSD microbiomes, and between the four time points during the first year of life. Microbiome-level relative reaction and subsystem abundances (Supplementary Data 6 and 7) as well as absolute reaction presence (Supplementary Data 8) were retrieved and statistically significantly different features were determined via Wilcoxon rank sum test, *p*-value < 0.05 after correction for false discovery rate (FDR) (“Methods”, Supplementary Data 9). Clear differences were found between microbiomes at different time points, whereby the relative abundances of 3076 of all 8185 reactions, the absolute presence of 1259 of all 8185 reactions, and the relative abundances of 85 of all 143 metabolic subsystems differed significantly over time (Table 1). Microbiomes at 5 days were lowest in functional diversity as demonstrated by reactions distinct in absolute presence across time points, with the number of present metabolic capabilities increasing throughout the first year of life (Fig. 2a). Hence, the modeling demonstrated that the metabolic capabilities present in infant microbiomes varied greatly during the first year of life. Increasing taxonomical diversity associated with the establishment of the gut microbiome during the first year of life^11^ was also associated with increasing functional diversity, or the number of unique reactions present, in the corresponding samples’ microbiome models.

**Table 1:** Number of features that differed significantly between microbiome models of vaginally delivered infants (VD) and those delivered through Cesarian section (CSD), as well as between infants at different time points

Dataset	Secretion capacity	Reaction abundance	Reaction presence	Subsystem abundance	Species-level metabolite contribution
vs. time points (all samples)	215(4)/854	3076(716)/8185	1259(912)/8185	85(9)/143	242(174)/3900
VD vs. CSD (5 days)	21(51)/295	2033(1207)/8185	275(332)/8185	65(11)/143	6(16)/3900
VD vs. CSD (1 month)	0(23)/295	277(827)/8185	80(276)/8185	0(0)/143	0(23)/3900
VD vs. CSD (6 months)	0(7)/295	0(472)/8185	0(243)/8185	0(1)/143	0(17)/3900
VD vs. CSD (1 year)	0(11)/295	0(705)/8185	0(20)/8185	0(37)/143	0(58)/3900

Shown are the number of features out of the total features that differed significantly after correction for multiple testing out of the total number of features present in at least one microbiome model. The number of initially significant features is shown in brackets. The tested features include net secretion potential (secretion capacity), relative abundance on reaction level, absolute reaction presence, relative abundance for reactions summarized by metabolic subsystem, and species to metabolite contributions.

### CSD gut microbiomes are depleted in metabolic capabilities

Performing a statistical analysis for VD and CSD microbiomes at each time point (Wilcoxon rank sum test, *p*-value < 0.05 after FDR correction) revealed that the highest number of statistically significant reactions and subsystems was present at 5 days with the abundances of 2033 reactions and 65 subsystems, and the absolute presence of 275 reactions differing significantly (Table 1). At 1 month of age, the relative abundance of 277 reactions and the absolute presence of 80 reactions were still significantly different between VD and CSD microbiomes (Table 1). Hence, the largest differences in reaction and pathway abundance between VD and CSD microbiomes were seen at the earliest stage of life. This was expected as the composition of the gut microbiome differed most between VD and CSD at this stage^11^. Compared with VD microbiomes, CSD microbiomes were clearly depleted in both relative abundance and absolute presence for a wide variety of reactions, as nearly all significantly differentially abundant reactions between VD and CSD were higher in VD (Fig. 2b, Supplementary Data 9). For instance, at 5 days, metabolic subsystems reduced in relative abundance in CSD microbiomes included drug and xenobiotics metabolism, fatty acid synthesis, glycerophospholipid metabolism, nucleotide interconversion, pentose and glucuronate interconversions, and glycolysis/gluconeogenesis (Supplementary Data 9a). Reactions belonging to the subsystems of starch and sucrose metabolism, O-glycan degradation, heparan sulfate degradation, HMO degradation, and plant polysaccharide degradation were also clearly depleted in CSD both in relative abundance and absolute presence (Supplementary Data 9a, b). The reduced abundance and absolute presence of reactions in starch and sucrose and O-glycan metabolism, and plant polysaccharide degradation still remained at 1 month of age (Supplementary Data 9a, b). Finally, while no reactions or subsystems were significant after correction for false discovery rate at 6 months and 1 year, the abundance of 472 and 705 reactions, respectively, was still initially significant at these time points (Table 1, Fig. 2b, Supplementary Data 9a, b). Taken together, reduced metabolic capabilities were seen in CSD compared to VD microbiomes at 5 days of life, which were still present to an extent at 1 month but largely disappeared at later stages of life.

### Community-level metabolic potential of the infant gut microbiome

Next, we evaluated through simulations how the sample-specific metabolic reactions translated into the microbiome communities’ total potential to synthesize and secrete metabolic products. For simulations, the microbiome models were appropriately contextualized with breast milk- or formula-feeding-based diets specific to each time point (Methods, Supplementary Data 4). The theoretical potential for biosynthesis of each secreted metabolite in each infant microbiome in mmol/person/day was subsequently computed (“Methods”, Supplementary Data 10). In total, 295 metabolites were predicted to be secreted by at least one infant microbiome. We determined the correlations between metabolite secretion and species-level abundances (Fig. S1). Several metabolites, including 1,2-propanediol and thiamin, were positively correlated with *Escherichia coli* but negatively correlated with Staphylococcus sp. (Fig. S1). Secondary bile acids correlated with *Eggerthella lenta*, as expected^45^.

**Fig. 3:**
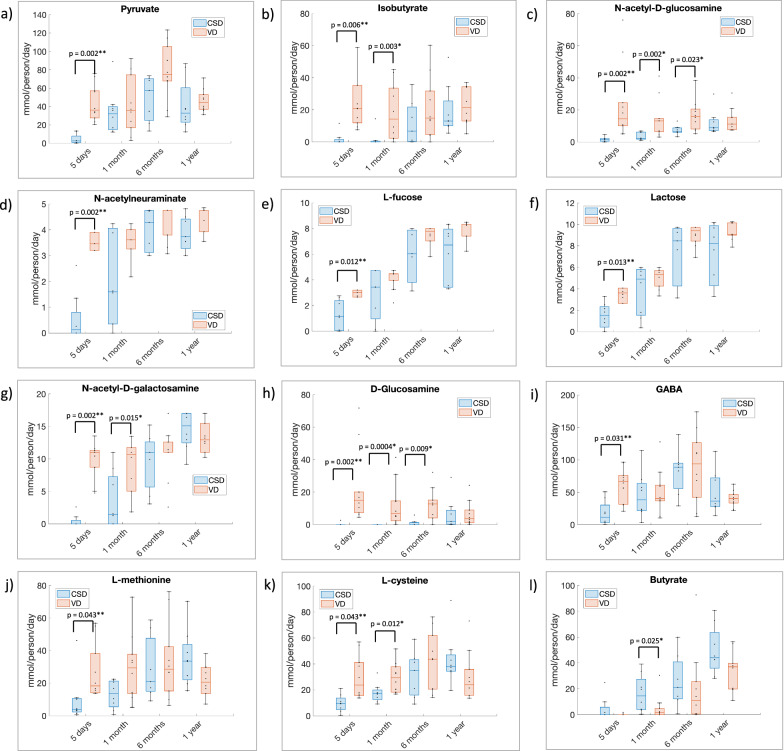
Predicted metabolite secretion capacity of infant microbiomes on the infant diet depicted by time point and birth mode. Shown are net secretion fluxes per microbiome sample in mmol/person/day for a subset of metabolites that were at least initially significantly different between vaginal delivery (VD) and Cesarian section delivery (CSD) for at least one-time point. **a** Pyruvate, **b** isobutyrate, **c** N-acetyl-D-glucosamine, **d** N-acetylneuraminate, **e** L-fucose, **f** lactose, **g** N-acetyl-D-galactosamine, **h** D-glucosamine, **i** GABA, **j** L-methionine, **k** L-cysteine, **l** butyrate. **p-value after correction for multiple testing, *initial *p*-value. All predicted net secretion fluxes are shown in Supplementary Data 10. *n* = 20 infant gut microbiome samples.

We next performed a statistical analysis (Wilcoxon rank sum test, *p*-value < 0.05 after FDR correction) for microbiome models’ maximal secretion capacity between the four time points and between VD and CSD microbiomes at each time point (“Methods”, Supplementary Data 11). As could be expected, the secretion capacity changed clearly during the first year of life with 215 metabolites differing significantly between the four time points (Table 1). Differences between the VD and CSD gut microbiomes were also observed. At 5 days of life, the secretion capacity for 21 metabolites was significantly different after correction for multiple testing (Table 1), all of which were lower in CSD. A further 51 metabolites were initially significant but not after correction for multiple testing (Table 1). At later time points, no metabolites were significantly different after correction for multiple testing, but 23, 7, and 11 metabolites were initially significant at 1 month, 6 months, and 1 year, respectively (Table 1). Metabolites depleted in CSD at 5 days included the fermentation products pyruvate and isobutyrate (Fig. 3a, b), Notably, CSD microbiomes at 5 days had a reduced capacity to secrete N-acetylglucosamine, N-acetylneuraminate, l-fucose, and lactose, all of which are components of HMOs^6^ (Fig. 3c–f). Moreover, CSD microbiomes were depleted in the capacity to secrete N-acetylgalactosamine and glucosamine (Fig. 3g, h), revealing an equally reduced capacity to degrade host-derived mucin o-glycans and glycosaminoglycans. The CSD microbiomes at 5 days also showed significantly reduced secretion of the neurotransmitter GABA (Fig. 3i), and the amino acids l-cysteine and -methionine, which are involved in the one-carbon metabolism and transulfuration pathways (Fig. 3j, k). In contrast, the predicted production of butyrate was initially significantly higher in CSD at 1 month (Fig. 3l). At 6 months, N-acetylglucosamine and d-glucosamine secretion were still initially significantly lower in CSD microbiomes (Fig. 3c, h). Hence, differences in metabolic potential in the microbiomes of CSD infants, though subtle, still remained at later stages in the first year of life, in agreement with previous functional analyses performed for the same cohort^11^. Finally, we evaluated the effect of antibiotics on net secretion capacity and found it had little impact as no metabolites were statistically significant after correction for multiple testing (Supplementary Data 11).

### Infant and maternal gut microbiomes differ in metabolic structure and function

Infant and adult gut microbiomes differ in their composition; for example, the infant gut has a higher relative abundance of bifidobacteria and streptococci^9^. For a subset of 13 mothers enrolled in the cohort used in this study, gut microbiome samples had been published previously^34^. Microbiome community models were built and interrogated for gut maternal samples as previously for infant gut microbiomes (“Methods”). We determined relative subsystem abundances that were statistically significantly different (Wilcoxon rank sum test, *p*-value < 0.05 after FDR correction) between maternal and infant gut (Supplementary Data 12). For instance, the gut microbiomes of infants up to 6 months were enriched in squalene and cholesterol synthesis, limonene and pinene degradation, primary amine metabolism, and glycerophospholipid metabolism (Fig. S2). Maternal gut microbiomes had a higher abundance in the nucleotide salvage pathway, tRNA charging, and chondroitin sulfate degradation (Fig. S2).

**Fig. 4:**
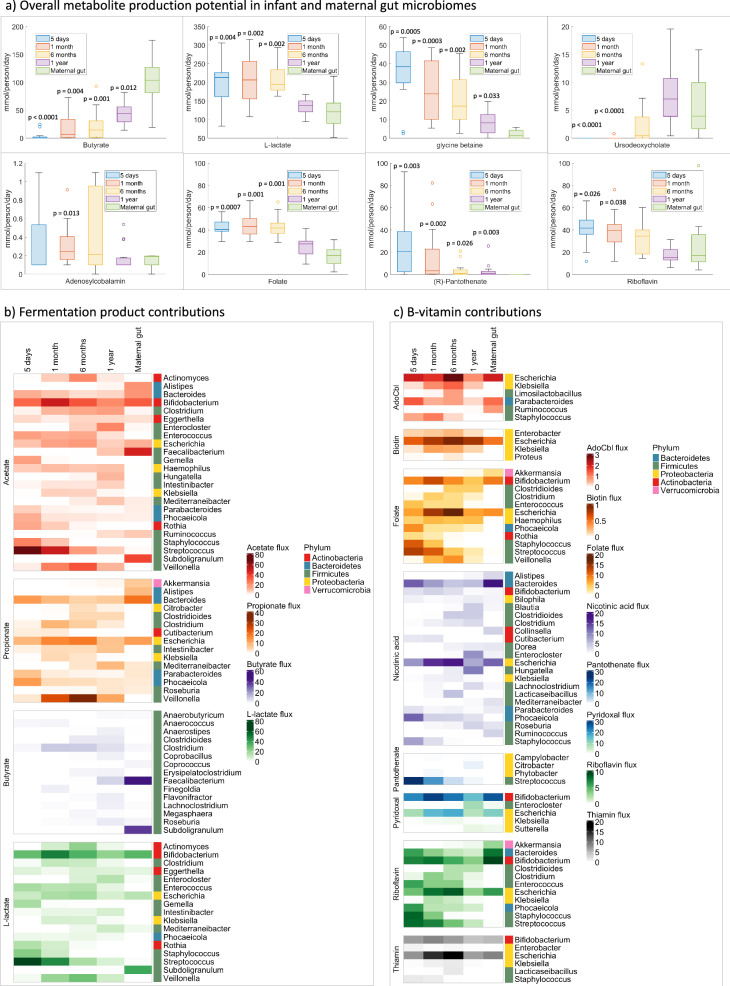
Features differing between infant and maternal gut microbiomes. **a** Predicted metabolite secretion capacity of infant microbiomes at the four-time points and maternal gut microbiomes on the Average European (AE) diet. Shown are net secretion fluxes per microbiome sample in mmol/person/day for a subset of metabolites that differed between adult and infant gut for at least one time point. Significant *p*-values after correction for multiple tests against maternal gut microbiome samples are indicated. All predicted net secretion fluxes on the AE diet are shown in Supplementary Data 13. **b**, **c** Taxon-metabolite contributions (mmol/person/day) on the AE diet for infant and maternal gut microbiomes. Species to metabolite contributions were averaged for all samples in each stratification group (infant gut microbiomes at all time points and maternal gut microbiomes) and then summarized on the genus level. Shown are contributions to **b** fermentation products, **c** B vitamins. All predicted microbe-metabolite contributions are shown in Supplementary Data 15. *N* = 20 infant and 13 maternal gut microbiome samples.

We then predicted for all samples the microbiome-wide potential to secrete metabolites on an Average European diet (Supplementary Data 13). For the 288 metabolites net secreted by at least one infant or maternal gut microbiome, a statistical analysis was performed (Wilcoxon rank sum test, *p*-value < 0.05 after FDR correction) (Methods, Supplementary Data 14). For instance, maternal gut microbiomes had a clearer higher potential to secrete butyrate, while infant gut microbiomes were enriched in the secretion of l-lactate (Fig. 4a). The infant’s gut had a higher secretion capacity for the primary amine glycine betaine, while the synthesis of secondary bile acids, e.g., ursodexycholate, was much lower infants than adults during the first months (Fig. 4a). Compared with the maternal gut, infant gut microbiomes also had enriched capability to secrete the B-vitamins cobalamin, folate, pantothenate, and riboflavin (Fig. 4a). Taken together, the microbiome-level potential to secrete metabolites including short-chain fatty acids, amines, bile acids, and B-vitamins differed between infant and maternal gut microbiomes.

Next, we aimed to predict the microbial taxa that could synthesize metabolites of interest in infant and maternal gut microbiomes. To this end, the taxon-level contributions to fermentation products and B-vitamins were computed (Supplementary Data 15, “Methods”) and are shown summarized on the genus level in Fig. 4b, c. According to our simulations, genera contributing to short-chain fatty acids changed during the first year of life and differed from adults (Fig. 4b). In infants, the genera Bifidobacterium, Streptococcus, and Veillonella were major acetate producers, while in adults, Faecalibacterium and Subdoligranulum were the highest producers (Fig. 4b). Faecalibacterium and Subdoligranulum were also the main butyrate producers in adults but not infants (Fig. 4b). Veillonella was a major propionate producer only in infants, while Akkermansia contributed to propionate mainly in adults (Fig. 4b). l-lactate, as expected, was synthesized mainly by infant microbial taxa such as Bifidobacterium, Streptococcus, and Veillonella (Fig. 4b).

Microbial production of B-vitamins was also predicted to differ between infant and maternal gut microbiomes (Fig. 4c). The genus Escherichia was a major contributor to biotin, cobalamin, folate, pyridoxal, riboflavin, and thiamin. Bifidobacterium was another major vitamin-producing genus and contributed to folate, pyridoxal, riboflavin, and thiamin. Other major producers included Bacteroides (nicotinic acid, riboflavin), Streptococcus (folate, pantothenate, riboflavin), and Staphylococcus (folate, riboflavin). The high B-vitamin biosynthesis capabilities of infant microbial taxa, namely Bifidobacterium and Streptococcus, explained the increased biosynthesis potential observed in the infant compared with the maternal gut microbiome (Fig. 4a).

## Discussion

Here, we aimed to investigate the impact of birth mode on the metabolic pathways and capabilities of infant gut microbiome throughout the first year of life. To this end, we have developed a computational workflow to build and interrogate species-specific community models of the gut microbiome from infants delivered vaginally and through the Cesarian section. First, we expanded a resource of genome-scale metabolic reconstructions of human microbes, AGORA2, with pathways for the degradation of human milk oligosaccharides. The resulting expanded reconstructions are freely available. Next, we built community metabolic models by mapping metagenomic data from the gut microbiomes of 20 infants at four time points during the first year of life. We predicted the present functions and metabolic capabilities of each gut microbiome and identified functions and metabolic fluxes that differed between infants delivered vaginally and through the Cesarian section as well as between time points. Finally, we built and interrogated community models for maternal gut microbiomes from the same cohort and identified functions and metabolic fluxes differing between infant and adult gut microbiomes. Our key results include that (i) at the earliest stages, CSD gut microbiomes were depleted in metabolic functions compared with VD gut microbiomes though these differences largely disappeared at later time points, (ii) the metabolic diversity, as indicated by the number of unique reactions, increased during the first year of life, (iii) the metabolic capabilities of infant and adult gut microbiomes were clearly distinct in their potential to synthesize fermentation products and B-vitamins and in taxa contributing most to these metabolites.

It is well established that the gut microbiomes of infants delivered vaginally and through the Cesarian section are distinct in the first months of life, though they become more similar over time^9^. Hence, there has been concern over the impact of the Cesarian section on the maturation of the developing infant gut microbiome^14^. Increases in Cesarian section may be linked to the increase in noncommunicable diseases such as obesity in the last decades, however, these associations are still unclear^14^. Our simulations predicted that though birth mode resulted in only subtle functional differences between VD and CSD at 1 year, it had a clear impact on the metabolic structure and capabilities of the gut microbiome during the first weeks of life, with a predicted lower capability to produce various metabolites in CSD microbiomes (Figs. 2–3). For instance, CSD microbiomes had a lower capacity to produce the neurotransmitter GABA at early time points (Fig. 3i), which plays a role in the gut–brain axis. Reduced GABA and pyruvate, but increased butyrate production at 5 months have been found in the fecal metabolome of infants at risk of autism^46^. Another study found higher fecal butyrate concentrations in CSD infants at 1 month^47^, also in agreement with our predictions (Fig. 3l).

We predicted the fermentation product profiles of infant and adult gut microbiomes and found they were distinct (Fig. 4a). Our prediction that butyrate production was low early on and increased in production later while l-lactate decreased after 6 months agrees with a recent cohort study^48^. The simulations further revealed that differences in fermentation products were due to the distinct composition of the infant gut microbiome, with higher abundances in bifidobacteria and facultative anaerobes, but lower abundances in the Faecalibacterium and Subdoligranulum genera compared with adult gut microbiomes (Fig. 4b, c). This finding agrees with the known composition of the developing infant gut microbiome^9^. In future studies, our predictions on metabolite secretion profiles could be validated further using fecal metagenomic and metabolomic data from the same infant cohort. Indeed, in a previous study, butyrate production predicted by microbiome models was validated against matched metabolomic data for a cohort of colorectal cancer patients and controls^49^. The modeling revealed species beneficial or deleterious for total butyrate production by the community^49^. A similar approach could be used to validate metabolite production predictions in infant microbiome communities for a cohort with matched metagenomic and metabolomic data.

Our simulations predicted that infant gut microbiomes had enriched biosynthesis potential for cobalamin, folate, pantothenate, and riboflavin (Fig. 4a). Specifically, Bifidobacterium species such as *B. longum* substantially contributed to folate, pyridoxal, riboflavin, and thiamin biosynthesis (Fig. 4c). Several Bifidobacterium strains are folate producers, as well as known probiotics, and supplementation with bifidobacteria, could increase folate levels in serum and feces^50^. Moreover, B-vitamin biosynthesis genes, including folate, nicotinic acid, and riboflavin, were enriched in infant gut-associated bifidobacteria such as *B. longum* subsp. infantis but absent in Bifidobacterium species of non-human origin^51^. Hence, the infant’s large intestinal microbiome may contribute to B-vitamin homeostasis in early life.

The potential of infant gut microbes to contribute to folate status in the host especially has implications for epigenetic regulation through DNA methylation^17,52^. Vitamin B12, which was also synthesized by microbes (Fig. 4a), equally plays a key role in epigenetic regulation through the synthesis of methionine, the immediate precursor of the universal methyl donor S-adenosylmethionine^53^. Modulation of metabolic programming through early-life exposures may result in long-lasting health outcomes, including obesity and metabolic syndrome according to the DOHaD hypothesis^14,17^. In addition, the vitamin B12 and folate status during pregnancy influence the risk of obesity and metabolic syndrome^17,54^. Notably, our simulations predicted that CSD microbiomes at 5 days had a lower potential to synthesize methionine (Fig. 3j), which is a key component in one-carbon metabolism, as well as a reduced abundance of 25 and two reactions involved in vitamin B12 and folate metabolism, respectively (Supplementary Data 9a). It has been shown that the gut microbiome substantially impacts DNA methylation^55^. Butyrate, which was predicted to be higher in CSD at 1 month (Fig. 3l), and lower in infant than in adult gut microbiomes (Fig. 4a), also modulates epigenetic regulation by inhibiting histone deacetylases^18^. For instance, butyrate inhibits the histone deacetylase SIRT1, which plays an important role in the regulation of fatty acid oxidation, inflammation, and insulin sensitivity^56^. Moreover, butyrate modulates host appetite and regulates metabolism by signaling to free fatty acid receptor 2 (FFAR2, GRP43) and free fatty acid receptor 3 (FFAR3, GRP41)^57^. Future studies may elucidate the impact of the infant gut microbiome on epigenetic regulation later in life, and potential links to noncommunicable diseases such as obesity and metabolic syndrome. It should be noted that while microbiome models can be readily integrated with the human host^58^, constraint-based modeling in its basic form does not account for regulatory mechanisms including epigenetics^59^. Hence, directly linking our predictions to epigenetic regulation in the host is not possible in the present study. However, recent works have integrated epigenetics into genome-scale models, e.g., histone acetylation and deacetylation^60^, methylation^61^, and posttranslational modification^62^. In future studies, metabolite production predicted by microbiome models could be integrated with human models accounting for epigenetic mechanisms such as acetylation or methylation.

Our study has several limitations. First, the sample size of the COSMIC cohort is relatively small with only 20 infants. Hence, the statistical power to distinguish features based on birth mode was limited, resulting in relatively few significantly different features after correction for multiple testing. Moreover, the modeled cohort included only few formula-fed infants^11^, hence, the impact of feeding mode could not be evaluated. Information on the type of formula, the composition of breast milk, or food frequency questionnaires for mothers, which could be used to further contextualize the models, were also not available. In future efforts, the modeling and analysis pipeline we have established in this work as a proof of concept could be applied to larger cohorts with available nutritional information to validate our predictions. Finally, the results of the modeling pipeline are subject to the general limitations of the constraint-based modeling approach, namely, the steady-state assumption which results in the prediction of fluxes rather than concentrations and a solution space rather than a single optimal flux distribution^38^, and the lack of kinetic and regulatory constraints^59^. Hence, the metabolite secretion predicted in the present work reflects the optimal production by the microbiome community given the present microbial species, reaction stoichiometry, and availability of dietary nutrients. Regulatory constraints that would limit the secretion of particular metabolites are presently not accounted for but could be included in future efforts^59^.

In summary, we have developed a pipeline for mechanistically modeling the infant gut microbiome. The pipeline identified differences in metabolic capabilities between infants by birth mode and between infants and adults. Modeling of mother-infant cohorts with available information on factors implicated in fetal programming, e.g., feeding mode, infant diet, maternal diet, maternal weight, and chemicals^63,64^ could provide insight into the influence of these exposures on the developing infant. For example, in a recent study, AGORA2 was used to build community models for a cohort of infant gut microbiomes and predict the effect of different food items in combination with breast milk on microbial growth and function^65^. Infant gut microbiomes could also be compared with samples from the same individuals later in childhood where available. Correlating infant gut microbiome samples with matched methylome data could provide insight into the impact of microbial metabolism on epigenetic regulation. Ultimately, computational modeling of the infant microbiome could improve our understanding of the links between microbial metabolism and infant health and development.

### Reporting summary

Further information on research design is available in the Nature Portfolio Reporting Summary linked to this article.

## Supplementary information

Supplementary Information

Description of Additional Supplementary Files

Supplementary Data 1

Supplementary Data 2

Supplementary Data 3

Supplementary Data 4

Supplementary Data 5

Supplementary Data 6

Supplementary Data 7

Supplementary Data 8

Supplementary Data 9

Supplementary Data 10

Supplementary Data 11

Supplementary Data 12

Supplementary Data 13

Supplementary Data 14

Supplementary Data 15

Reporting Summary

## Data Availability

The 250 AGORA2 reconstructions expanded with HMO degradation pathways, and the additional 289 genome-scale reconstructions created in this study are available at https://zenodo.org/uploads/14238419^66^. The sequencing data used in this study that had been generated previously^11^ are available under BioProject accession number PRJNA595749. The source data for Fig. 2 can be found in Supplementary Data 6 and 8. The source data for Fig. 3 can be found in Supplementary Data 10 and for Fig. 4 in Supplementary Data 13 and 15.

## Code availability

Scripts and input data that enable reproducing the generated reconstructions, microbiome simulations, and analyses, including figures, are available at https://github.com/almut-heinken/ngereSysBio/tree/main/infantMicrobiome^67^.
