# Supplementary material for: Personalized modeling of gut microbiome metabolism throughout the first year of life
Source: Commun Med (Lond). 2024 Dec 30;4:281. doi: 10.1038/s43856-024-00715-4 (PMC11686179; doi:10.1038/s43856-024-00715-4)
Supplement: Supplementary file 1 — Supplementary Information [file 43856_2024_715_MOESM1_ESM.pdf]

Supplementary information for:

Personalized modeling of gut microbiome metabolism throughout the first year of life

Rola Shaaban[1#], Susheel Bhanu Busi[2,3], Paul Wilmes[2,4], Jean-Louis Guéant[1,5], and Almut Heinken[1*]

[1]Inserm UMRS 1256 NGERE, University of Lorraine, Nancy, France

[2]Luxembourg Centre for Systems Biomedicine, University of Luxembourg, Esch-sur-Alzette, Luxembourg

[3]UK Centre for Ecology and Hydrology, Wallingford, Oxfordshire, United Kingdom

[4]Department of Life Sciences and Medicine, Faculty of Science, Technology and Medicine, University of Luxembourg, Esch-sur-Alzette, Luxembourg

[5]National Center of Inborn Errors of Metabolism, University Regional Hospital Center of Nancy, Nancy, France

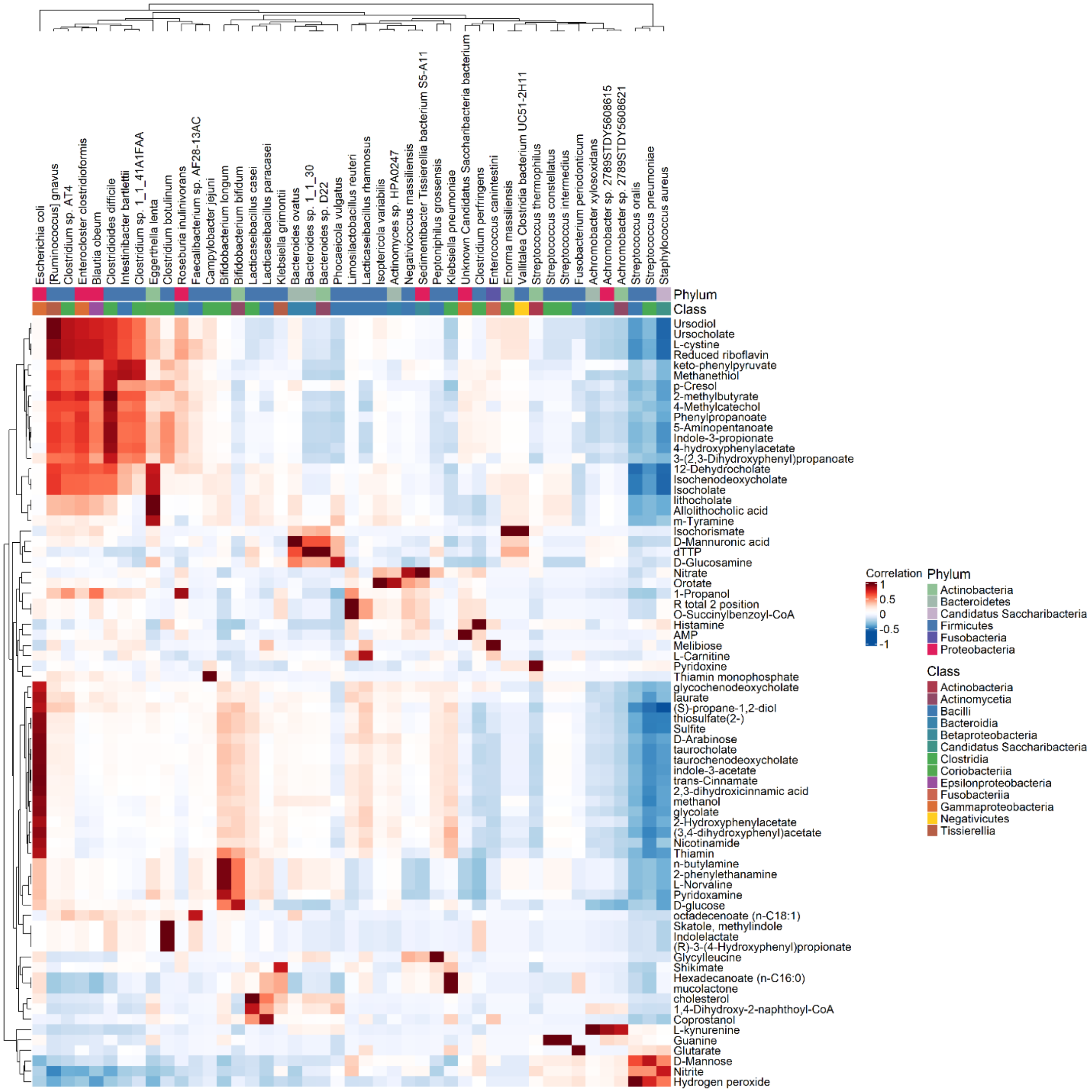

Figure S1: Spearman correlations between computed microbiome-level net secretion fluxes and relative species abundances. Rows indicate net secreted metabolites and columns indicate species annotated by class and phylum. Shown are metabolites that had a correlation of at least +/- 0.8 for at least one species.

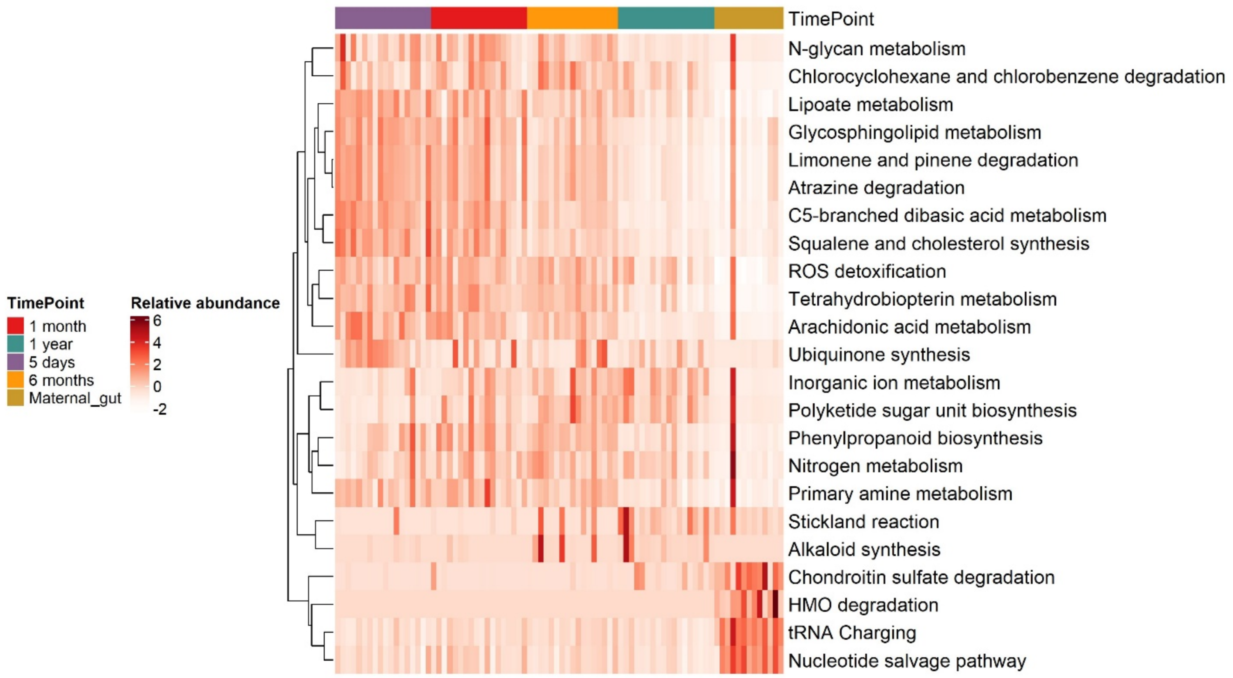

Figure S2: Subsystems differing in relative abundance between infant and maternal gut microbiomes. Shown are subsystems that were highly significantly different (p-value <0.001 after correction for multiple testing) between maternal gut and infant gut for at least one time point.
