# Supplementary material for: Personalized modeling of gut microbiome metabolism throughout the first year of life
Source: Commun Med (Lond). 2024 Dec 30;4:281. doi: 10.1038/s43856-024-00715-4 (PMC11686179; doi:10.1038/s43856-024-00715-4)
Supplement: Supplementary file 2 — Description of Additional Supplementary Files [file 43856_2024_715_MOESM2_ESM.pdf]

Description of Additional Supplementary Files

File name- Supplementary Data 1:

File description- Human milk oligosaccharide degradation in AGORA2 with supporting references.

File name- Supplementary Data 2:

File description- Metabolites and reactions formulated for the human milk oligosaccharide degradation module.

File name- Supplementary Data 3:

File description- Features of the 289 additionally generated genome-scale reconstructions.

File name- Supplementary Data 4:

File description- Features of the personalized infant and maternal gut microbiome community models generated in this study.

File name- Supplementary Data 5:

File description- Community-level reaction abundance in infant and maternal gut microbiome models.

File name- Supplementary Data 6:

File description- Community-level subsystem abundance in infant and maternal gut microbiome models.

File name- Supplementary Data 7:

File description- Reactions that differed statistically significantly by birth mode summarized by metabolic subsystem.

File name- Supplementary Data 8:

File description- Constraints used to simulate the infant diets.

File name- Supplementary Data 9:

File description- Net secretion capacity on the infant diets in infant gut microbiome models.

File name- Supplementary Data 10:

File description- Statistical analysis of net secretion capacity on the infant diets by birth mode, antibiotics use, and time point.

File name- Supplementary Data 11:

File description- Statistical analysis of subsystem abundance between maternal and infant gut microbiomes at different time points.

File name- Supplementary Data 12:

File description- Net secretion capacity on the Average European diet in infant and maternal gut microbiome models.

File name- Supplementary Data 13:

File description- Statistical analysis of net secretion capacity on the Average European diet between maternal and infant gut microbiomes at different time points.

File name- Supplementary Data 14:

File description- Species-level contributions to total metabolite secretion in infant and maternal gut microbiome models.

File name- Supplementary Data 15:

File description- the taxon-level contributions to fermentation products and B-vitamins were computed (Supplementary Data 15, Methods)
